# Supplementary material for: An improved biometric stress monitoring solution for working employees using heart rate variability data and Capsule Network model
Source: PLoS One. 2024 Dec 17;19(12):e0310776. doi: 10.1371/journal.pone.0310776 (PMC11651562; doi:10.1371/journal.pone.0310776)
Supplement: Attachment — Submitted filename: Response_Letter_Biometric_Stress.pdf [file pone.0310776.s001.pdf]

Submission ID: PONE-D-24-18697

Article Title: An Improved Biometric Stress Monitoring Solution For Working Employees Using Heart Rate Variability Data and Capsule Network Model

June 26, 2024

Dear Editors,

First of all, we would like to extend our sincere gratitude to all Editors for allowing a re-submission of our manuscript, with an opportunity to address the reviewer's comments.

We have done our best to modify the paper to reflect all the comments made by the anonymous reviewers. These changes are fully addressed in this reply letter. For clarity, we have marked our responses in blue.

Please find the enclosed revised version of the paper along with the summary of the reviewer's comments. For clarity, our responses have been written in blue color. whenever we copy text from the manuscript, it is given in red color.

REVIEWER 1

Reviewer # 1 (Concern # 1)

Strengths:
- Comprehensive literature review and context setting.
- Clear description of the methodology and experimental setup.
- Impressive accuracy rates and robust validation through k-fold cross-validation.
- Comparative analysis with state-of-the-art models.

Respected reviewer, Thank you very much for your appreciation.

Reviewer # 1 (Concern # 2)

The introduction could be more concise with a clearer focus on the study's objectives.

Respected reviewer, we have added the following lines to address this concern:

In today's high-pressure work environments, effective stress management is essential for maintaining employee health and productivity. Traditional stress assessment methods, often based on self-reports, are subjective and unreliable. Heart Rate Variability (HRV) provides a robust, objective measure of stress by reflecting autonomic nervous system activity. This paper presents an advanced biometric stress monitoring solution utilizing HRV data and a Capsule Network model, which outperforms conventional CNNs in recognizing complex patterns. Our approach aims to deliver real-time, accurate stress detection, facilitating timely interventions and fostering a healthier workplace.

Reviewer # 1 (Concern # 3)

The related work section, while thorough, could benefit from a summary table or diagram.

Thank you very much for your suggestions. Respected reviewer, we have added a summary table at the end of the related work section:

Table 1: Summary of state-of-the-art related work.

Ref	Classifier	Dataset	Performance
[9]	Hybrid Calibration method, Generic models, Person-specific models	SWELL, WESAD	95.02% Hybrid Calibration Method
[4]	NB, DT, SVM	Self-collected (voice and heart rate data)	88.53% SVM
[8]	SMA	WESAD	86% stress recognition, 98% stress detection
[3]	kNN, LSVM, RBFSVM, DT, RF, MLP, ADA, NB, QDA, Gaussian Process, Deep ECGNet	Kwangwoon university	87.39% Deep ECGNet
[10]	RF, DT, SVM,KNN, DeepER Net (CNN and LSTM)	16 different datasets	83.9% DeepER Net
[1]	LOF, MLP	SWELL, WESAD	99.04% MLP on WASAD, 88.64% MLP on SWELL
[5]	KNN, SVM, Bayesian Approaches, ANN, Classification tress	SWELL	90% SVM
[12]	ANN	SWELL	96.09%
[11]	SFSS, RSFS, SFFS+GDA, SBS, MERNN	WESAD	92.43% MERNN
[2]	RF, SVM, LR	Empatica E4 device self-collected dataset	76.5% RF

Reviewer # 1 (Concern # 4)

More detailed explanations of preprocessing and feature extraction methods in the methodology section.

Respected reviewer, we have updated the dataset section by adding pre-processing and segmentation details. In pre-processing and segmentation, the conversion of raw signals from interval-based signals is done. The contextual factors are considered for 25 seconds using a sliding window of 5 seconds while physiological factors are 60 seconds (1 minute) using a sliding window of 20 seconds. The updated section is:

0.1 Dataset

The study's dataset is taken from Kaggle, a website well-known for its benchmark dataset. The "Biometrics for stress monitoring" dataset is publicly accessible. This dataset integrates electrodermal activity (EDA) and heart rate variability (HRV) information taken from the SWELL and WESAD datasets [6, 7]. It is structured into three main directories and sub-directories for efficient organization and analysis. The "interim" directory contains transformed intermediate data, including ground truth labels (Labels), raw EDA signals (eda), and inter-beat intervals (rri) derived from ECG signals. The "processed" directory houses files computed from the intermediate data, aiding in analysis procedures. In pre-processing and segmentation, the conversion of raw signals from interval-based signals is done. The contextual factors are considered for 25 seconds using a sliding window of 5 seconds while physiological factors are 60 seconds (1 minute) using a sliding window of 20 seconds. Within the "final" directory, there are two subdirectories: "results," which contains specific published outcomes from the aforementioned paper, and "datasets," which contain combined train, test, and validation data used for model creation. These subdirectories are further detailed in the paper [9]. This structured setup facilitates easy access and utilization of the dataset for research and

development in stress prediction models.

Reviewer # 1 (Concern # 5)
Inclusion of additional performance metrics (precision, recall, F1-score) in the results.

Respected reviewer, we have address accuracy, precision, recall, and F1-score in all results tables. As well as we have added the Area under curve (AUC) evaluation parameter.

Table 2: Swell dataset (multi-class, 3 classes).

Models	Accuracy	Precision	Recall	F1 score	AUC
Xception	86.35	82.55	83.52	82.53	85.32
EfficientNetB4	83.05	82.38	81.57	82.53	82.29
CNN	85.86	86.58	86.89	86.78	84.63
VGG19	90.09	83.82	84.78	83.71	90.02
ResNET	84.53	83.36	84.54	83.47	83.39
MobileNet	91.62	89.87	89.56	89.70	91.52
InceptionV3	90.70	89.52	89.95	89.79	88.26
CapsNets	92.76	91.78	92.43	92.59	92.14

Table 3: Experimental results on the WESAD dataset (multi-class, 3 classes).

Models	Accuracy	Precision	Recall	F1 score	AUC
Xception	89.35	92.54	93.52	92.53	89.20
EfficientNetB4	87.85	92.40	91.57	92.53	85.56
CNN	90.86	93.58	94.90	93.78	88.74
VGG19	92.91	93.82	94.76	93.72	91.47
ResNET	94.53	93.36	94.54	93.47	94.29
MobileNet	95.63	94.87	96.56	95.48	92.23
InceptionV3	95.63	94.52	96.95	95.53	94.87
CapsNets	96.76	97.73	98.43	97.89	95.92

Reviewer # 1 (Concern # 6)
Expanded discussion on practical applications, challenges, and ethical considerations.

Respected reviewer, we have added the following subsections to address this concern:

0.2 Discussion on practical applications, challenges, and ethical considerations

Biometric stress monitoring using heart rate variability (HRV) data combined with advanced machine learning models like the Capsule Network (CapsNet) offers a promising solution for enhancing employee well-being and productivity. This document discusses the practical applications, challenges, and ethical considerations associated with implementing such a system.

0.2.1 Practical Applications

1. Workplace Health and Safety: Implementing a biometric stress monitoring solution can significantly enhance workplace health and safety. By continuously monitoring employees' stress levels through HRV data, employers can identify individuals who may be experiencing high levels of stress and take proactive measures to alleviate it. This can help prevent workplace accidents and improve overall employee well-being.

2. Productivity Optimization: Stress has a direct impact on productivity. By identifying periods of high stress, employers can optimize work schedules, provide timely breaks, and implement stress-relief interventions. This can lead to a more productive and efficient workforce.

3. Personalized Health Interventions: With real-time data on stress levels, personalized health interventions can be provided to employees. For example, stress management workshops, mental health counseling, and relaxation techniques can be tailored to the needs of individuals based on their stress patterns.

4. Remote Work and Flexibility: In the era of remote work, monitoring stress levels can provide valuable insights into the well-being of employees who are not physically present in the office. This can help in maintaining a healthy work-life balance and ensuring that remote employees are not overburdened.

0.2.2 Challenges

1. Data Privacy and Security: The collection and processing of biometric data raise significant privacy and security concerns. Ensuring that employees' HRV data is securely stored and only accessible to authorized personnel is crucial. Compliance with data protection regulations like GDPR is necessary to avoid legal repercussions.

2. Integration with Existing Systems: Integrating the stress monitoring system with existing workplace health and management systems can be complex. Ensuring compatibility and seamless data flow between different platforms requires careful planning and execution.

0.2.3 Ethical Considerations

1. Informed Consent: Employees must be fully informed about the nature of the biometric stress monitoring system, the type of data collected, and how it will be used. Obtaining explicit consent is crucial to ensure ethical compliance.

2. Data Transparency: Employees should have access to their own stress data and be informed about how it is being used. Transparency in data handling builds trust and ensures ethical use of biometric information.

3. Supportive Interventions: The primary goal of stress monitoring should be to support employees rather than to surveil them. Interventions should be aimed at improving well-being and should include resources and support for stress management.

REVIEWER 2

Reviewer # 2 (Concern # 1)

This paper is well-written; however, a few improvements are needed:
English Corrections: Please review the text for any grammatical or typographical errors.

Thank you very much for pointing out this lack in paper. Respected reviewer, we have improved the sentence structure, removal of grammatical mistakes, and typos from the paper.

Reviewer # 2 (Concern # 2)

Additional Details: Please provide more information about the results. For instance, specify which kernel was used for the SVM and include the TensorFlow version, among other relevant details.

Respected reviewer, we have added hyperparameter details of all learning models with all experimental setup information. The hyperparameter details of all learning models are shown in Table 4

Table 4: Hyperparameters for Various Transfer Learning Models

Model	Learning Rate	Batch Size	Epochs	Optimizer
Xception	0.001	32	50	Adam
EfficientNetB4	0.001	32	50	Adam
CNN	0.01	64	100	SGD
VGG19	0.0001	32	50	Adam
ResNet	0.001	32	50	Adam
MobileNet	0.001	32	50	Adam
InceptionV3	0.001	32	50	Adam
Capsule Network	0.0005	32	50	Adam

0.3 Experimental setup

The Python 3.8 programming environment is used to conduct the research. The study's experimental setting includes the computer language Python 3.8, (Scikit learn version Version 1.5.0 and TensorFlow version r2.15), RAM capacity available (8GB DDR4), operating system type (64-bit Windows 11), CPU specifications are Intel Core i7 with a processor frequency at about 2.8 GHz which belongs to the 7th generation and an Nvidia GTX1060 GPU. This information is relevant for comprehending the technical characteristics of the research setting and the computational resources employed in this study.

References

[1] M. Albaladejo-González, J. A. Ruipérez-Valiente, and F. Gómez Mármol. Evaluating different configurations of machine learning models and their transfer learning capabilities for stress detection using heart rate. *Journal of Ambient Intelligence and Humanized Computing*, 14(8):11011–11021, 2023.

[2] S. Campanella, A. Altaleb, A. Belli, P. Pierleoni, and L. Palma. A method for stress detection using empatica e4 bracelet and machine-learning techniques. *Sensors*, 23(7):3565, 2023.

[3] B. Hwang, J. You, T. Vaessen, I. Myin-Germeys, C. Park, and B. T. Zhang. Deep ecgnet: An optimal deep learning framework for monitoring mental stress using ultra short-term ecg signals. *TELEMEDICINE and e-HEALTH*, 24(10):753–772, 2018.

[4] T. Y. Kim, L. Měsíček, and S. H. Kim. Modeling of child stress-state identification based on biometric information in mobile environment. *Mobile Information Systems*, 2021:1–13, 2021.

[5] S. Koldijk, M. A. Neerincx, and W. Kraaij. Detecting work stress in offices by combining unobtrusive sensors. *IEEE Trans. Affect. Comput.*, 9:227–239, 2018.

[6] S. Koldijk, M. A. Neerincx, and W. Kraaij. Detecting work stress in offices by combining unobtrusive sensors. *IEEE Trans. Affect. Comput.*, 9(2):227–239, 2018.

[7] P. W. Kraaij, M. S. Koldijk, and M. M. Sappelli. The swell knowledge work dataset for stress and user modeling research. `https://doi.org/10.17026/dans-x55-69zp`, 2014.

[8] K. Lai, S. N. Yanushkevich, and V. P. Shmerko. Intelligent stress monitoring assistant for first responders. *IEEE Access*, 9:25314–25329, 2021.

[9] K. Nkurikiyeyezu, A. Yokokubo, and G. Lopez. The effect of person-specific biometrics in improving generic stress predictive models. *arXiv preprint arXiv:1910.01770*, 2019.

[10] W. Seo, N. Kim, S. Kim, C. Lee, and S. M. Park. Deep ecg-respiration network (deeper net) for recognizing mental stress. *Sensors*, 19(13):3021, 2019.

[11] M. Vellaisamy and L. I. Freitas. Detection of human stress using optimized feature selection and classification in ecg signals. *Mathematical Problems in Engineering*, 2023, 2023.

[12] R. Walambe, P. Nayak, A. Bhardwaj, and K. Kotecha. Employing multimodal machine learning for stress detection. *J. Healthc. Eng.*, 2021:9356452, 2021.
