# Supplementary material for: An improved biometric stress monitoring solution for working employees using heart rate variability data and Capsule Network model
Source: PLoS One. 2024 Dec 17;19(12):e0310776. doi: 10.1371/journal.pone.0310776 (PMC11651562; doi:10.1371/journal.pone.0310776)
Supplement: Attachment — Submitted filename: Response_Letter_Mashael_Plos_One_Biometric_Stress.pdf [file pone.0310776.s002.pdf]

Submission ID: PONE-D-24-18697R1

Article Title: An Improved Biometric Stress Monitoring Solution For Working Employees Using Heart Rate Variability Data and Capsule Network Model

July 20, 2024

Dear Editors,

First of all, we would like to extend our sincere gratitude to all Editors for allowing a re-submission of our manuscript, with an opportunity to address the reviewer's comments.

We have done our best to modify the paper to reflect all the comments made by the anonymous reviewers. These changes are fully addressed in this reply letter. For clarity, we have marked our responses in blue.

Please find the enclosed revised version of the paper along with the summary of the reviewer's comments. For clarity, our responses have been written in blue color. whenever we copy text from the manuscript, it is given in red color.

Academic Editor Comments

Academic Editor (Concern # 1)

Recent existing survey works must be improved by including the overall analysis as a separate table.

Respected Academic Editor, We have added this summary table to address your concern about summarizing the overall recent existing works. The added table is 1.

Academic Editor (Concern # 2)

Author needs to include some effective validation techniques of the proposed system

Respected Academic Editor, We have included accuracy, precision, recall, f1 score, and area under curve five evaluation parameters to assess the working of our proposed system. The evaluation parameters provide every detail about a system that is doing classification tasks. Furthermore, we have added a cross-validation technique to address this concern.

Academic Editor (Concern # 3)

Please address the reviewer's comments properly and proofread for whole manuscript.

Respected Academic Editor, We have carefully addressed the reviewer 2 minor concern about adding % sign with evaluation parameters labeling on top of the table. We also proofread the complete paper to avoid any human error and typos mistakes.

REVIEWER 1

Table 1: Literature review summary table.

Ref	Classifier	Dataset	Performance
[7]	Hybrid Calibration method, Generic models, Person-specific models	SWELL, WESAD	95.02% Hybrid Calibration Method
[4]	NB, DT, SVM	Self-collected (voice and heart rate data)	88.53% SVM
[6]	SMA	WESAD	86% stress recognition, 98% stress detection
[3]	kNN, LSVM, RBFSVM, DT, RF, MLP, ADA, NB, QDA, Gaussian Process, Deep ECGNet	Kwangwoon university	87.39% Deep ECGNet
[8]	RF, DT, SVM,KNN, DeepER Net (CNN and LSTM)	16 different datasets	83.9% DeepER Net
[1]	LOF, MLP	SWELL, WESAD	99.04% MLP on WASAD, 88.64% MLP on SWELL
[5]	KNN, SVM, Bayesian Approaches, ANN, Classification tress	SWELL	90% SVM
[10]	ANN	SWELL	96.09%
[9]	SFSS, RSFS, SFFS+GDA, SBS, MERNN	WESAD	92.43% MERNN
[2]	RF, SVM, LR	Empatica E4 device self-collected dataset	76.5% RF

Reviewer # 1 (Concern # 1)
All of my comments have been addressed.

Respected reviewer, Thank you very much for your appreciation and accepting this work in its current form.

REVIEWER 2

Reviewer # 2 (Concern # 1)
This paper is very well written. All the comments were addressed by the author however, please re-check the data representation in the tables. For instance, Table no 6 has Accuracy, it should be like " Accuracy (%)".

First of all thank you so much for accepting this paper and pointing out this minor lack in paper. Respected reviewer, we have added the % sign in the tables where necessary.

References

[1] M. Albaladejo-González, J. A. Ruipérez-Valiente, and F. Gómez Mármol. Evaluating different configurations of machine learning models and their transfer learning capabilities for stress detection using heart rate. *Journal of Ambient Intelligence and Humanized Computing*, 14(8):11011–11021, 2023.

[2] S. Campanella, A. Altaleb, A. Belli, P. Pierleoni, and L. Palma. A method for stress detection using empatica e4 bracelet and machine-learning techniques. *Sensors*, 23(7):3565, 2023.

[3] B. Hwang, J. You, T. Vaessen, I. Myin-Germeys, C. Park, and B. T. Zhang. Deep ecgnet: An optimal deep learning framework for monitoring mental stress using ultra short-term ecg signals. *TELEMEDICINE and e-HEALTH*, 24(10):753–772, 2018.

[4] T. Y. Kim, L. Měsíček, and S. H. Kim. Modeling of child stress-state identification based on biometric information in mobile environment. *Mobile Information Systems*, 2021:1–13, 2021.

[5] S. Koldijk, M. A. Neerincx, and W. Kraaij. Detecting work stress in offices by combining unobtrusive sensors. *IEEE Trans. Affect. Comput.*, 9:227–239, 2018.

[6] K. Lai, S. N. Yanushkevich, and V. P. Shmerko. Intelligent stress monitoring assistant for first responders. *IEEE Access*, 9:25314–25329, 2021.

[7] K. Nkurikiyeyezu, A. Yokokubo, and G. Lopez. The effect of person-specific biometrics in improving generic stress predictive models. *arXiv preprint arXiv:1910.01770*, 2019.

[8] W. Seo, N. Kim, S. Kim, C. Lee, and S. M. Park. Deep ecg-respiration network (deeper net) for recognizing mental stress. *Sensors*, 19(13):3021, 2019.

[9] M. Vellaisamy and L. I. Freitas. Detection of human stress using optimized feature selection and classification in ecg signals. *Mathematical Problems in Engineering*, 2023, 2023.

[10] R. Walambe, P. Nayak, A. Bhardwaj, and K. Kotecha. Employing multimodal machine learning for stress detection. *J. Healthc. Eng.*, 2021:9356452, 2021.
